# Dacarbazine depletes the ovarian reserve in mice and depletion is enhanced with age

**DOI:** 10.1038/s41598-018-24960-5

**Published:** 2018-04-25

**Authors:** Amy L. Winship, Monika Bakai, Urooza Sarma, Seng H. Liew, Karla J. Hutt

**Affiliations:** Ovarian Biology Laboratory, Biomedicine Discovery Institute, Department of Anatomy and Developmental Biology, Monash University, Melbourne, Australia

## Abstract

Dacarbazine is commonly administered for the treatment of cancers prevalent in reproductive age females. However, investigations of off-target effects of dacarbazine on the ovary are limited. We assessed the impact of dacarbazine on the ovarian reserve of primordial follicles, essential for fertility. Eight week and 6 month old C57BL/6 J mice were administered with dacarbazine or saline on day (d)0 and d7, then sacrificed after 12 hours (h), or 14d (n = 4–5/group). Follicle numbers, follicle density, serum AMH and corpora lutea were quantified and estrous cyclicity monitored. In reproductively young mice, dacarbazine did not affect primordial follicle numbers at 12 h, but resulted in a 36% reduction at 14d (p < 0.05). Dacarbazine-mediated primordial follicle depletion was accelerated with age, with a 24% (p < 0.05) and 36% (p < 0.01) reduction at 12 h and 14d. Follicle density remained unchanged between treatment groups at either age. Dacarbazine depleted antral follicles at 14d (p < 0.05), at both ages. Despite partial reduction of antral follicles, serum AMH, estrous cyclicity and corpora lutea (indicative of ovulation) remained unchanged between treatment groups, at both ages. Importantly, diminished ovarian reserve can result in premature ovarian insufficiency and infertility, thus, fertility preservation options should be considered for young female patients prior to dacarbazine treatment.

## Introduction

The ovary contains a finite number of germ cells (oocytes) within primordial follicles. Primordial follicles give rise to all mature oocytes and are thus required to sustain the female fertile lifespan^1^. Primordial follicles remain dormant in the ovary, until being activated to undergo folliculogenesis and ovulation, or alternatively, atresia^2^. With advancing age, the ovarian reserve of primordial follicles diminishes, until menopause ensues. Primordial follicles are highly susceptible to genotoxic stress and their depletion is accelerated in response to anti-cancer treatments, including γ-irradiation and chemotherapy (reviewed^3–5^). As a consequence, premature ovarian insufficiency, early onset menopause and infertility are major side effects for reproductively aged females receiving anti-cancer treatment.

Recent advances in the early detection and treatment of cancer have significantly improved the 5-year survival rate of patients^6^, increasing the importance of understanding the adverse long term effects of chemotherapy on the ovary (reviewed^4^). Currently, about 5% of cancer diagnoses occur in women who are under the age of 40^6^. The degree of chemotherapy-induced ovarian damage has been described to be highly dependent on the drug class, as well as the age at treatment, with oocyte depletion occurring at an increased rate with advancing age^7–9^. Although mice do not undergo menopause, ovarian aging, reproductive decline and loss of ovarian endocrine function are similar between humans and mice^10^. Surprisingly though, the comparative effects of chemotherapy on the ovary between reproductively young and older mice have never been investigated.

Of the chemotherapeutic agents with known gonadotoxic potential, it is well-established that classical alkylators such as cyclophosphamide cause primordial follicle depletion in the mouse^11^ and human ovary^12^. Dacarbazine is a non-classical alkylating agent, routinely administered as a single agent to treat malignancies common in women of reproductive age, including melanoma, sarcoma and neuroblastoma. Dacarbazine is also frequently used in combination with adriamycin (doxorubicin), bleomycin, and vinblastine (ABVD) for the treatment of Hodgkin’s lymphoma^13^. Dacarbazine impairs cell division by causing DNA double-strand breaks and is referred to as a non-classical alkylating agent because its activity requires processing in the liver by microsomal metabolism, yielding methylating intermediates^14^.

The effects of single agent dacarbazine administration on the ovary are limited to one report in mice. Previously, it was shown that one intravenous low dose (100 μg) of dacarbazine led to a combined reduction of 16% of follicle density after 14 days (d)^15^. However, the frequency and concentration of the dose of dacarbazine used was not clinically applicable. Additionally, the study classified primordial (non-growing) and primary (growing) follicles together, and did not quantify absolute follicle numbers, making it difficult to interpret the direct effects of dacarbazine on the ovarian reserve of primordial follicles.

While studies of dacarbazine-induced ovarian damage are limited, there are numerous, yet conflicting reports on the impacts of the commonly administered dacarbazine-containing combined chemotherapy regimen, ABVD, on the ovary^16–20^. ABVD induced temporary amenorrhea, but only occasionally resulted in premature ovarian insufficiency in one small study of 65 women^16^. In a larger study of 2127 women, there was no increase in the rate of early-onset menopause in ABVD patients versus women that received alkylating agents alone (excluding dacarbazine), but non-treatment controls were not analysed^17^. More recently, McLaughlin *et al*. investigated follicle density in ovarian tissue biopsies from women treated with ABVD and found a higher density of primordial follicles compared to an untreated, or combined vincristine, etoposide, prednisone, doxorubicin (OEPA) and cyclophosphamide, vincristine, prednisone, dacarbazine (COPDAC) treated cohort of women^19^. This unexpected finding was contradicted by a study from Sonigo *et al*., in which the number of cumulus-oocyte complexes recovered, as well as the total number of matured oocytes vitrified was significantly lower in 22 patients that previously received AVBD treatment at least 2 years prior, versus 44 age-matched women that received no chemotherapy^20^. This finding suggests a potential reduction in the size ovarian reserve or, compromised ability of primordial follicles to develop through to maturity following ABVD. Despite these inconsistent findings and paucity of experimental studies, it is the current clinical recommendation that the dacarbazine-containing ABVD protocol has a low-risk of causing ovarian toxicity and that fertility preservation should not be considered^21^.

In order to better understand the risk of dacarbazine to female fertility, in this study we aimed to comprehensively investigate its effects on the ovary by determining the absolute number and density of primordial follicles and all other follicle classes. Serum AMH concentrations, estrous cycling and histological markers of ovarian follicle atresia, proliferation, DNA damage and ovarian fibrosis were also examined. For these studies we used a mouse model, as accurate analyses of primordial follicle numbers following exposure to chemotherapy are not possible in women. Additionally, since the extent of chemotherapy-induced ovarian damage is age-dependent, we examined the effects of dacarbazine on the ovary in both reproductively young and older mice.

## Materials and Methods

### Animals, treatments and tissue collection

Female 8 week old (reproductively young; peak fertility) and 6 month old (reproductively older; still fertile) C57BL/6 mice were housed in a temperature-controlled high barrier facility (Monash University ARL), with free access to food and water, under a 12 h light-dark cycle. All animal procedures and experiments were performed in accordance with the NHMRC Australian Code of Practice for the Care and Use of Animals and approved by the Monash Animal Research Platform Animal Ethics Committee. Mice (n = 5/age/treatment/time point) were weighed prior to intravenous tail vein injection with 130 mg/kg of dacarbazine (Sigma-Aldrich), or saline vehicle control on day d0 and d7. The conversion of this dose regimen is relevant to humans^22^ (for example a single cycle of ABVD usually includes 2 doses of 375 mg/m^2^ dacarbazine, but other dacarbazine regimens vary)^13^ and previous administration of 100–200 mg/kg dacarbazine was not reported to cause morbidity or mortality in mice^23^. Mice were not estrous cycle staged prior to treatment, although, following the second injection, estrous cycling was monitored by vaginal cytology for 14 consecutive days, as detailed previously^24^. Slides containing vaginal smears were stained with Rapid Diff Stain Kit (Australian Biostain) and categorised based on the cell types present as previously described^24^. Mice were humanely killed either 12 h or 14d following final treatment, followed by terminal cardiac puncture to collect peripheral blood. Serum was obtained, post coagulation and separated by centrifugation, then stored at −80 °C before use. Mice were weighed at necropsy and ovaries were harvested; one fixed in 10% (vol/vol) neutral buffered formalin solution for 24 h and paraffin embedded, and the other fixed in Bouin’s solution for 24 h before being processed into hydroxyethyl methacrylate resin (Technovit 7100; Kulzer and Co.).

### Follicle counts

Unbiased stereology is considered the best-practice method for the quantification of cells in tissue sections^25^ and was used to quantify follicle numbers (n = 4–5/age/treatment/time point). Resin embedded ovaries were serially sectioned at 20 μm with a RM2165 microtome (Leica Microsystems) and stained with periodic acid-Schiff and haematoxylin. Stereological follicle counts were performed as published^26^. Briefly, we used a ×100 oil immersion objective on a BX50 microscope (Olympus), mounted with an Autoscan stage (Autoscan Systems Pty Ltd), controlled by StereoInvestigator software (Version 11.06.02, MBF Bioscience 2015). Every third section was counted and primordial, transitional and primary follicle numbers were determined by multiplying the raw counts of oocytes sampled (Q−) by all three sampling fractions (1/f1, 1/f2 and 1/f3). Importantly, we only counted follicles in which the oocyte nucleus was visible and the standardised distance between sections ensures that follicles are not counted twice. The total number of healthy and atretic secondary and antral follicles in each ovary was estimated in every ninth section under a light microscope (Nikon). Follicles were counted if the oocyte nucleus was present and classified as atretic if ≥10% of granulosa cells appeared pyknotic, or if they contained a degenerating oocyte, indicated by the presence of eosinophilia, irregular oolemma or a fragmented germinal vesicle^27,28^. Total follicle numbers were obtained by multiplying the raw counts of oocytes sampled (Q−) by 9 to correct for the sections not counted. The number of corpora lutea were determined by direct counting of every sixth section encompassing the entire ovary. Adjacent sections were evaluated to ensure each corpora lutea was only counted once.

### Follicle density

Total follicle numbers (oocyte present) were counted in every ninth resin section using the Provis AX70 Widefield Microscope (Olympus). Cross-sectional tissue area was measured using CellSens Software (Olympus). Follicle density (n = 4–5/age/treatment/time point) was reported as average number of follicles per area (mm^2^).

### Ovarian Volume

Estimation of total ovarian volume (n = 4–5/age/treatment/time point) was performed according to the Cavalieri principle as previously described^29^. Briefly, a test system of grid points was overlaid on the image of the tissue at ×10 magnification using a stereology system and software as above, and repeated for every sixth section of the whole ovary. Volume was calculated using the following equation: *V* = *a(p)****∙****d****∙****tΣP*_*i*_ where, v is the volume; a(p) is the area associated with each grid point; d is the distance between two consecutive sections; t is the section thickness and ∑Pi is the sum of all points counted in each section.

### Anti-Müllerian hormone ELISA

Serum AMH concentrations (n = 4–5/age/treatment/time point) were determined using the AMH Gen II ELISA (Beckman Coulter) according to the manufacturer’s instructions. Standard curve calibrators, low and high positive controls (Beckman Coulter) and serum samples were assayed in duplicate and absorbance measured using the ClarioStar microplate reader (BMG Labtech).

### TUNEL assay

Parrafin embedded ovaries were serially sectioned (5 μm) and mounted on glass slides. The Apop Tag Peroxidase *In Situ* Apoptosis Detection Kit (Millipore) was used to detect apoptosis according to the manufacturer’s instructions. Sections were counterstained with haematoxylin. Images were captured on the Provis AX70 Widefield microscope (Olympus). Three to five sections per ovary per animal (n = 5/age/treatment) were analysed at the D7 + 12 h time point. Follicles were classified as positive if the oocyte and/or ≥2 granulosa cells were positive and expressed as a proportion (%) of total follicles.

### Immunohistochemistry

Three to five ovarian tissue sections (5 μm) from saline or dacarbazine treated mice at d7 + 12 h (n = 5/age/treatment) were systematically selected to provide representative samples from the whole ovary and then deparaffinized in histolene, before being rehydrated in a series of graded ethanols. Antigen retrieval was performed by microwaving the sections for 10 min in 0.01 M sodium citrate (pH 6). Endogenous peroxidases were quenched for 30 min in 0.3% H_2_O_2_, and non-specific binding of antibodies was blocked by incubating the sections for 1 h with 10% normal goat serum. Sections were incubated with primary antibody against Phospho-Histone H3 (Phospho S10) (1:500; Abcam #ab5176), Cleaved Caspase-3 (D175) (1:200; Cell Signaling Technology #9661 S) and Phospho-Histone (γ)H2AX (Ser139) (1:100; Cell Signaling Technology #9718) overnight at 4 °C and later with biotinylated goat antibody against rabbit IgG (1:500; Vector Laboratories) for 1 h at room temperature. Sections were incubated for 30 min with avidin–biotin peroxidase complex (Vector Laboratories). Peroxidase activity was visualised using 3,3′-diaminobenzidine and sections were counterstained with haematoxylin. Tissues stained with the primary antibody omitted were used as negative controls. Whole ovarian section (×10 magnification) and high power images (×40 magnification) were captured on the Provis AX70 Widefield microscope (Olympus). Positive follicles (positive staining in the oocyte and/or ≥2 granulosa cells) were expressed as a proportion (%) of total follicles.

### Picrosirius red staining

Tissue sections were deparaffinized in histolene, rehydrated in a series of graded ethanols, then immersed in Picro-Sirius red staining solution (0.1% w/v) comprising Sirius Red F3B (Sigma-Aldrich) in a saturated aqueous solution of picric acid (Sigma-Aldrich) for 1 h at room temperature. Slides were washed four times in 0.5% glacial acetic acid for 7 min per wash. Tissues were rapidly dehydrated in 100% ethanol, cleared in histolene and mounted with DPX. The area of positive stained ovarian tissue was quantified as previously reported^30^. Briefly, whole tissue section images were captured on the DotSlide system at ×20 objective using an XC10 camera (Olympus). ImageJ was used to quantify the area of positive staining above a threshold that was set based on the staining in the oldest animal. This threshold was kept constant for all images analysed. Four tissue sections per ovary per animal at d7 + 12 h were analysed (n = 4–5/age/treatment).

### Statistical Analysis

Data are presented as mean ± SEM and statistical analysis was performed using GraphPad Prism Software. Normally distributed data were analysed by student’s t-test to compare two groups and ANOVA with Tukey’s post-hoc test to compare multiple groups. Differences were considered significant when p < 0.05.

### Data availability

All data generated or analysed during this study are included in this published article (and its Supplementary Information files).

## Results

### Dacarbazine depletes the ovarian reserve of primordial follicles and enhances antral follicle atresia in reproductively young mice

**Figure 1:**
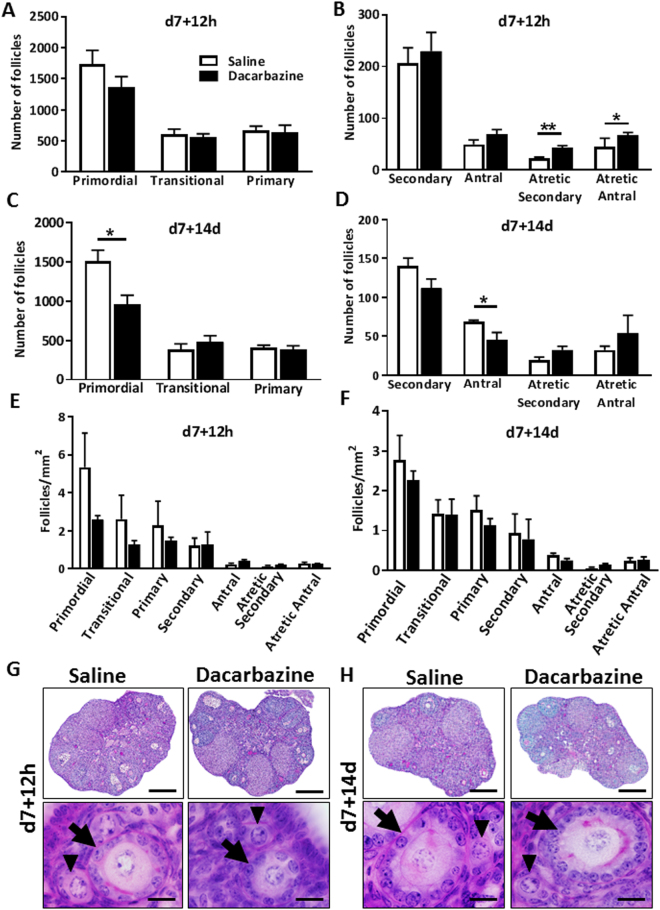
Female 8 week old C57BL/6 mice were treated with saline control, or 130 mg/kg dacarbazine on day (d)0 and 7 and ovaries harvested either 12 hours (h), or 14 d following final treatment. The number of primordial, transitional and primary follicles were quantified in saline or dacarbazine treated ovaries at (**A**) d7 + 12 h and (**C**) d7 + 14d. Healthy secondary, healthy antral, atretic secondary and atretic antral follicles were quantified at (**B**) d7 + 12 h and (**D**) d7 + 14d. Follicle density of all follicle classes was assessed at (**E**) d7 + 12 h and (**F**) d7 + 14d. Data are mean ± SEM; t-test, *p < 0.05, **p < 0.01; n = 4 mice/group (d7 + 12 h), or n = 5 mice/group d7 + 14d. Photomicrographs of PAS stained tissue sections highlight normal ovarian morphology between treatment groups at both (**G**) d7 + 12 h and (**H**) d7 + 14d. No morphological differences were observed in primordial (arrow head) and primary (arrow) follicles between treatment groups, at either time point. Scale bars = 200 µm (top panel); 20 µm (lower panel).

Follicle numbers were quantified in 8 week old mouse ovaries either 12 h, or 14d following the final administration of dacarbazine, or saline control. At 12 h, dacarbazine had no effect on primordial, transitional, primary (Fig. 1A), healthy secondary, or antral follicle numbers (Fig. 1B). Both atretic secondary (p < 0.01) and atretic antral follicles were significantly increased in response to dacarbazine compared to control (p < 0.05) (Fig. 1B). At 14d, primordial follicles were significantly depleted by 36% in response to dacarbazine treatment (saline 1505 ± 142 vs. dacarbazine 957 ± 119 follicles/ovary, p < 0.05) (Fig. 1C). Healthy antral follicles were also significantly reduced by dacarbazine (p < 0.05) (Fig. 1D), while there were no effects on other follicle classes. We found no changes in follicle density in all follicle classes in response to dacarbazine treatment versus control at 12 h, or 14d after the final dose (Fig. 1E,F). Despite changes in follicle numbers in response to dacarbazine administration (Fig. 1A–D), there were no gross morphological changes to the ovary structure, or surviving primordial and primary follicles, when compared to saline control (Fig. 1G,H). Dacarbazine treatment had no impact on total body weight (Supp. Figure 1).

### Dacarbazine depletes the ovarian reserve of primordial follicles and enhances antral follicle atresia in reproductively aged mice

**Figure 2:**
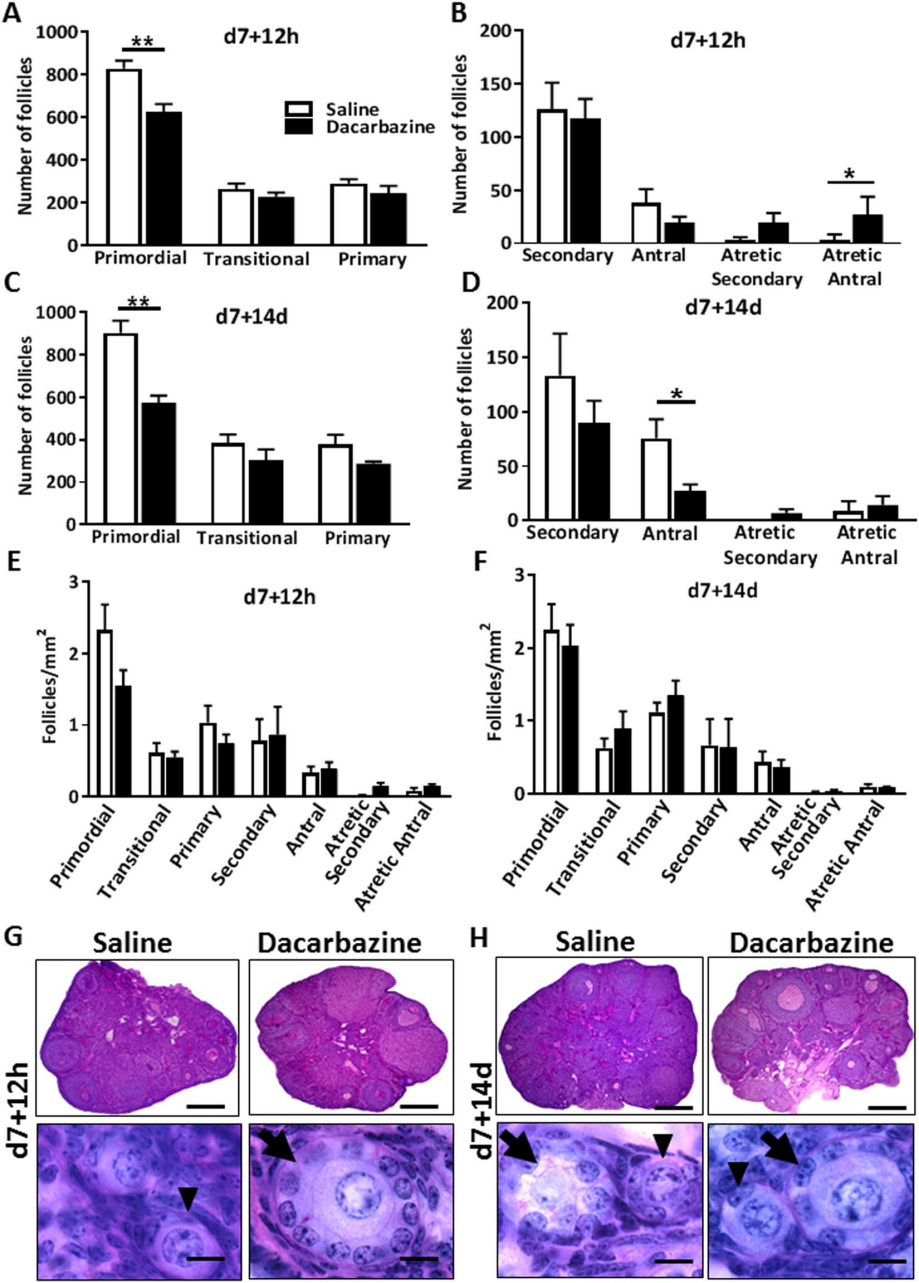
Female 6 month old C57BL/6 mice were treated with saline control, or 130 mg/kg dacarbazine and ovaries collected at d7 + 12, or d7 + 14d. The number of primordial, transitional and primary follicles were quantified in saline or dacarbazine treated ovaries at (**A**) d7 + 12 h and (**C**) d7 + 14d. Healthy secondary, healthy antral, atretic secondary and atretic antral follicles were quantified at (**B**) d7 + 12 h and (**D**) d7 + 14d. Follicle density of all follicle classes was assessed at (**E**) d7 + 12 h and (**F**) d7 + 14d. Data are mean ± SEM; t-test, *p < 0.05, **p < 0.01; n = 5 mice/group. PAS staining highlighted normal ovarian morphology at both (**G**) d7 + 12 h and (**H**) d7 + 14d. No morphological differences were observed in primordial (arrow head) and primary (arrow) follicles between treatment groups, at either time point. Bars = 200 µm (top pannel); 20 µm (lower pannel).

Six month old female mice were administered with dacarbazine, or saline vehicle control and follicle numbers determined at 12 h or 14d after the second dose. Dacarbazine significantly reduced primordial follicle numbers by 24% at 12 h following final treatment (saline 830 ± 37 vs. dacarbazine 628 ± 35 follicles/ovary, p < 0.01) (Fig. 2A). There were no changes in transitional, primary (Fig. 2A), healthy secondary, healthy antral, or atretic secondary follicle numbers between groups (Fig. 2B), although atretic antral follicles were significantly increased in response to dacarbazine treatment versus control at 12 h (p < 0.05) (Fig. 2B). At 14d following final treatment, primordial follicles were significantly depleted by 36% in response to dacarbazine (saline 904 ± 56 vs. dacarbazine 575 ± 33 follicles/ovary, p < 0.01) (Fig. 2C). Healthy antral follicles were also significantly reduced by dacarbazine (p < 0.05) (Fig. 2D), with no effects on other follicle classes. There were no differences in follicle density amongst all follicle classes, between treatment groups at both 12 h and 14d (Fig. 2E,F). PAS staining highlighted normal ovarian and follicle morphology in both treatment groups, at both time points (Fig. 2G,H). Dacarbazine treatment had no impact on total body weight (Supp. Figure 1).

### Dacarbazine-mediated depletion of the ovarian reserve of primordial follicles is enhanced with age in mice

**Figure 3:**
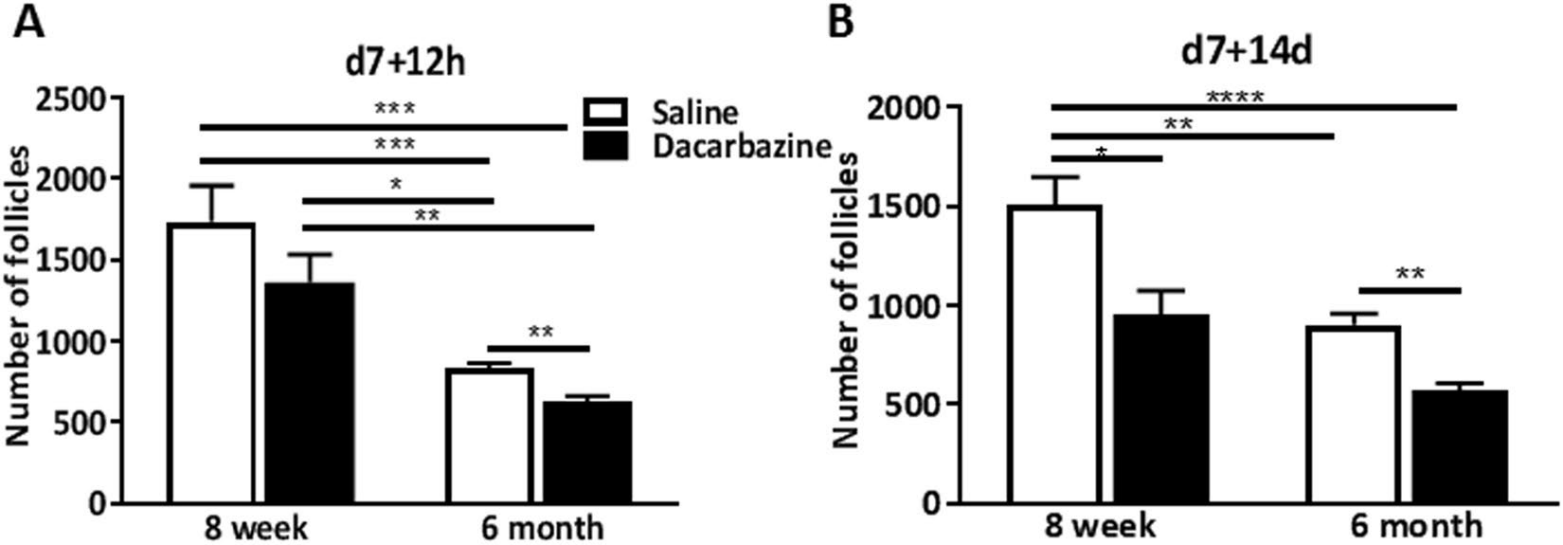
The effect of dacarbazine versus saline control on absolute primordial follicle numbers was compared between 8 week reproductively young mice and 6 month reproductively aged mice at (**A**) d7 + 12 h and (**B**) d7 + 14d. Data are mean ± SEM; ANOVA with Tukey’s post-hoc test, *p < 0.05, **p < 0.01, ***p < 0.001, ****p < 0.0001; n = 4–5/group.

Multiple comparisons were performed to examine the effects of age on dacarbazine-mediated primordial follicle loss. We observed a natural loss of primordial follicle numbers with age in saline control treated mice of 52% at d7 + 12 h (8 week saline 1735 ± 223 vs. 6 month saline 830 ± 37 follicles/ovary, p < 0.001) and 40% at d7 + 14d (8 week saline 1505 ± 142 vs. 6 month saline 904 ± 56 follicles/ovary, p < 0.01) (Fig. 3A,B). Dacarbazine accelerated this proportion of follicle loss in reproductively older mice versus control young mice by 64% at d7 + 12 h (8 week saline 1505 ± 142 vs. 6 month dacarbazine 628 ± 35 follicles/ovary, p < 0.001) and 62% at d7 + 14d (8 week saline vs dacarbazine 575 ± 33 follicles/ovary, p < 0.001) (Fig. 3A,B).

### Dacarbazine does not alter serum AMH, ovulation, or estrous cycling in mice

**Figure 4:**
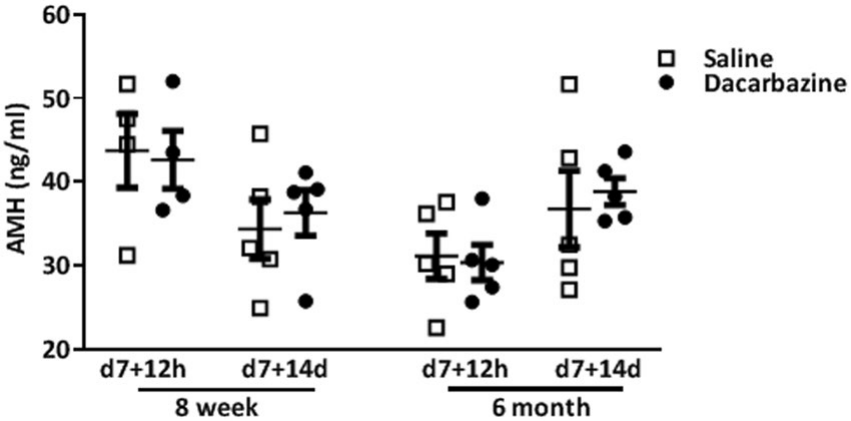
AMH serum concentrations of 8 week old and 6 month old saline or dacarbazine treated mice were quantified by ELISA. Data are mean ± SEM; t-test; n = 4–5/group.

AMH is primarily secreted from the granulosa cells of growing pre-antral follicles, which includes primary and secondary follicles, and is used clinically as a marker of the follicle reserve and to predict patient responses to chemotherapy (reviewed^5^). To investigate the impact of dacarbazine treatment on AMH levels, serum was collected 12 h or 14d following final treatment in reproductively young and older mice. There were no differences in serum AMH concentrations between saline, or dacarbazine treated mice, at either 12 h, or 14d post-treatment, in both 8 week and 6 month old mice (Fig. 4).

**Figure 5:**
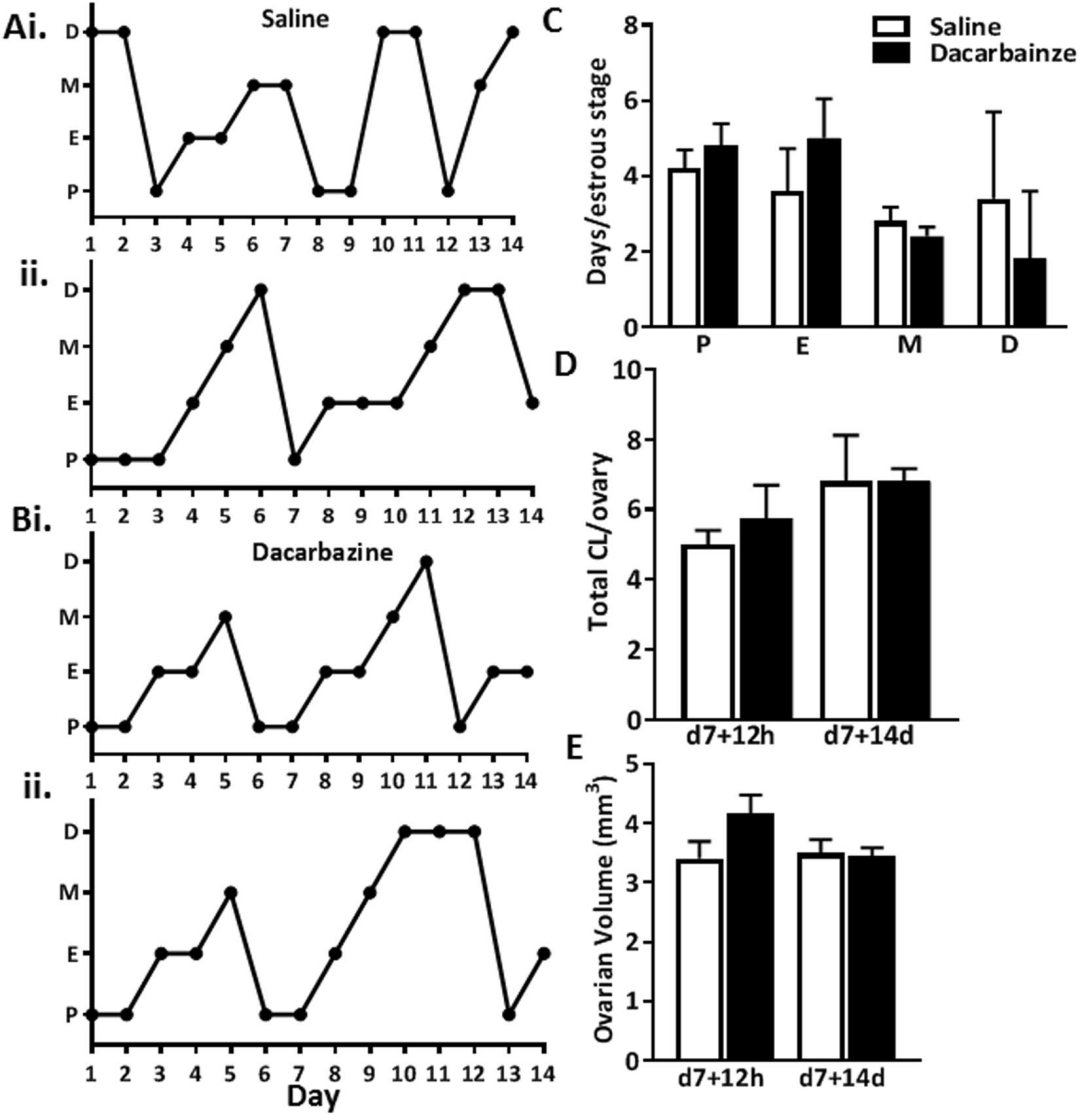
Estrous cycling was monitored by vaginal cytology for a 14 day period following final treatment in 8 week old mice (n = 5/group). Representative plots depict the estrous cycles of two individual (**Ai**,**ii**) saline control and (**Bi**,**ii**) dacarbazine treated mice. Vertical axes represent the phase of estrous cycle (P, proestrus; E, estrus; M, metestrus; D, diestrus). (**C**) The number of days spent in each stage of the estrous cycle was quantified for all saline and dacarbazine treated mice (n = 5/group). (**D**) Total number of corpora lutea (CL) per ovary and (**E**) ovarian volume were quantified in 8 week old saline and dacarbazine treated mice at d7 + 12 h and d7 + 14d. Data are mean ± SEM; t-test; n = 4 mice/group d7 + 12 h, or n = 5 mice/group d7 + 14d.

**Figure 6:**
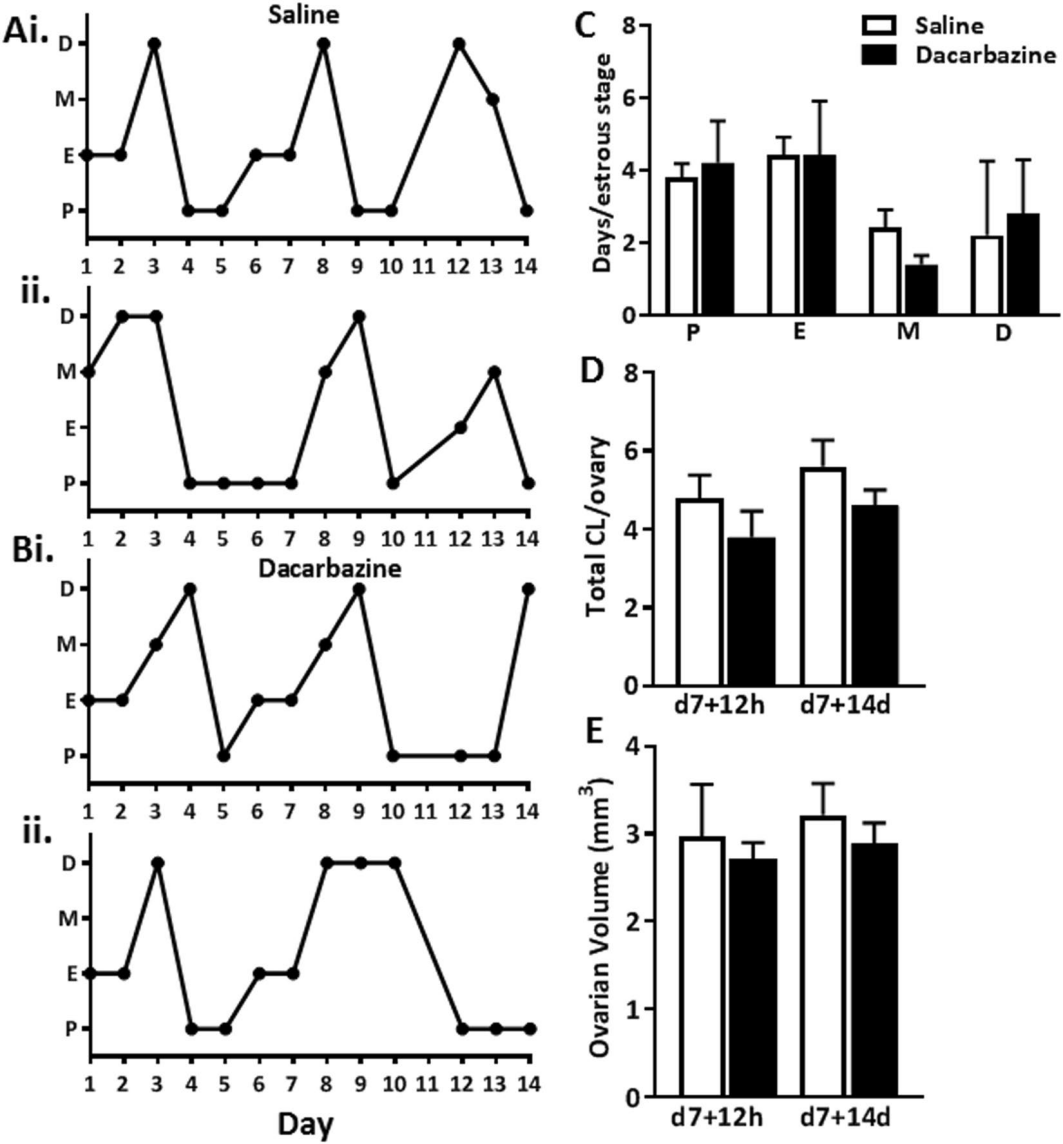
Estrous cycling was monitored by vaginal cytology for a 14 day period following final treatment in 6 month old mice (n = 5/group). Representative plots depict the estrous cycles of two individual (**Ai**,**ii**) saline control and (**Bi**,**ii**) dacarbazine treated mice. Vertical axes represent the phase of estrous cycle (P, proestrus; E, estrus; M, metestrus; D, diestrus). (**C**) The number of days spent in each stage of the estrous cycle was quantified for all saline and dacarbazine treated mice (n = 5/group). (**D**) Total number of corpora lutea (CL) per ovary and (**E**) ovarian volume were quantified in 6 month old saline and dacarbazine treated mice at d7 + 12 h and d7 + 14d. Data are mean ± SEM; t-test; n = 5 mice/group. Estrous cycling was monitored by vaginal cytology for a 14 day period following final treatment in 6 month old mice (n = 5/group).

From the examination of vaginal cytology, all saline and dacarbazine treated mice progressed through all stages of the estrous cycle, suggesting regular cyclicity (Fig. 5A–C). The presence of corpora lutea (CL) are indicative of ovulation. We found no differences in the number of CL between dacarbazine and saline treated mice at 12 h, or 14d following final treatment in 8 week old females (Fig. 5D). Given the large size of CL, their presence can dictate ovarian volume. In support, we found no differences in ovarian volume between treatment groups, at either time point (Fig. 5E). Similarly, in reproductively aged 6 month old female mice, dacarbazine did not impact on estrous cyclicity (Fig. 6A–C), ovulation, as indicated by CL number (Fig. 6D), ovarian volume (Fig. 6E).

### Dacarbazine increases antral follicle atresia in mice, but does not alter proliferation, DNA damage, or ovarian fibrosis

**Figure 7:**
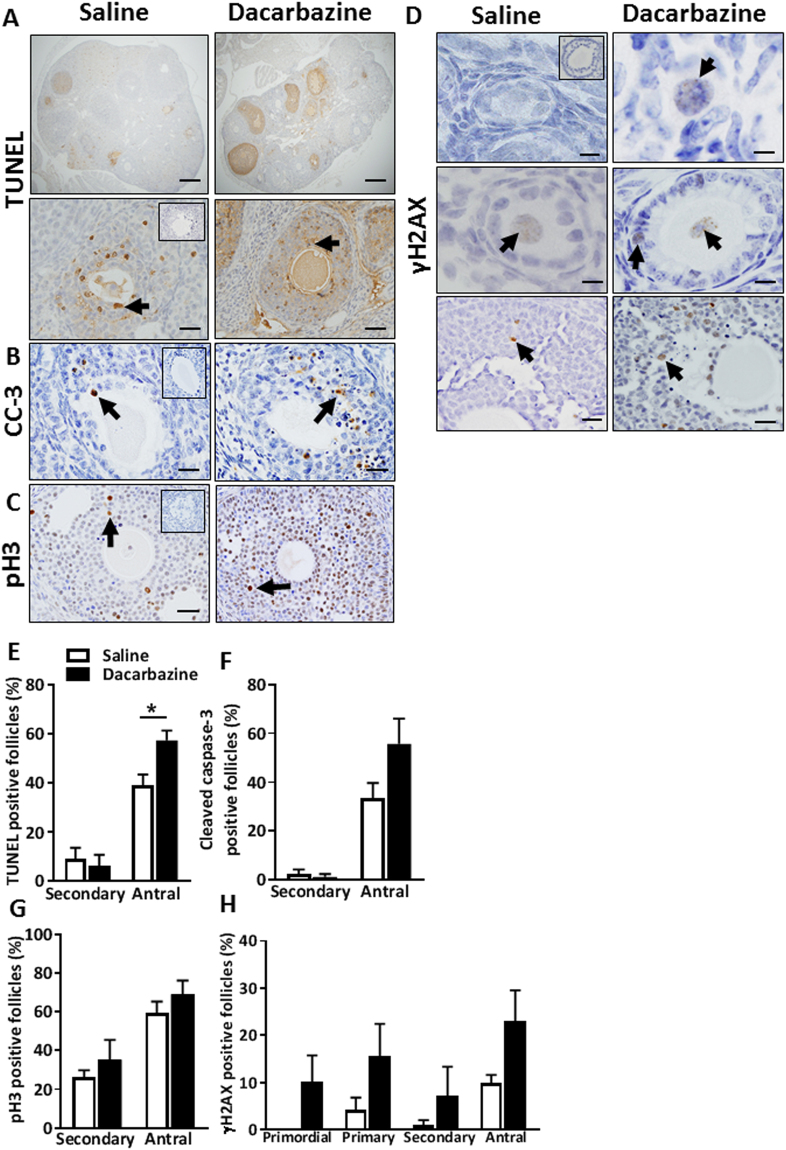
Analysis of follicle atresia, proliferation and DNA damage in 8 week old saline, or dacarbazine treated mouse ovaries at d7 + 12 h. Representative images of whole ovarian sections (upper panels) and positive follicles (brown staining, arrows, lower panels) stained for (**A**) TUNEL. Scale bars = 200 μm (upper panels) and 50 μm (lower panels). (**B**) Representative images of cleaved caspase-3 (CC-3) (Scale bars = 50 μm), (**C**) phospho-histone H3 (pH3) (Scale bars = 50 μm), and (**D**) γH2AX immunostained ovarian sections. Scale bars = 20 μm (upper panels) and 50 μm (middle and lower panels). Insets are negative controls. Quantification of the proportion (%) of positive stained follicles for (**E**) TUNEL, (**F**) cleaved caspase-3, (**G**) phospho-histone H3 and (**H**) γH2AX. Data are mean ± SEM; t-test; *p < 0.05, n = 5/group.

**Figure 8:**
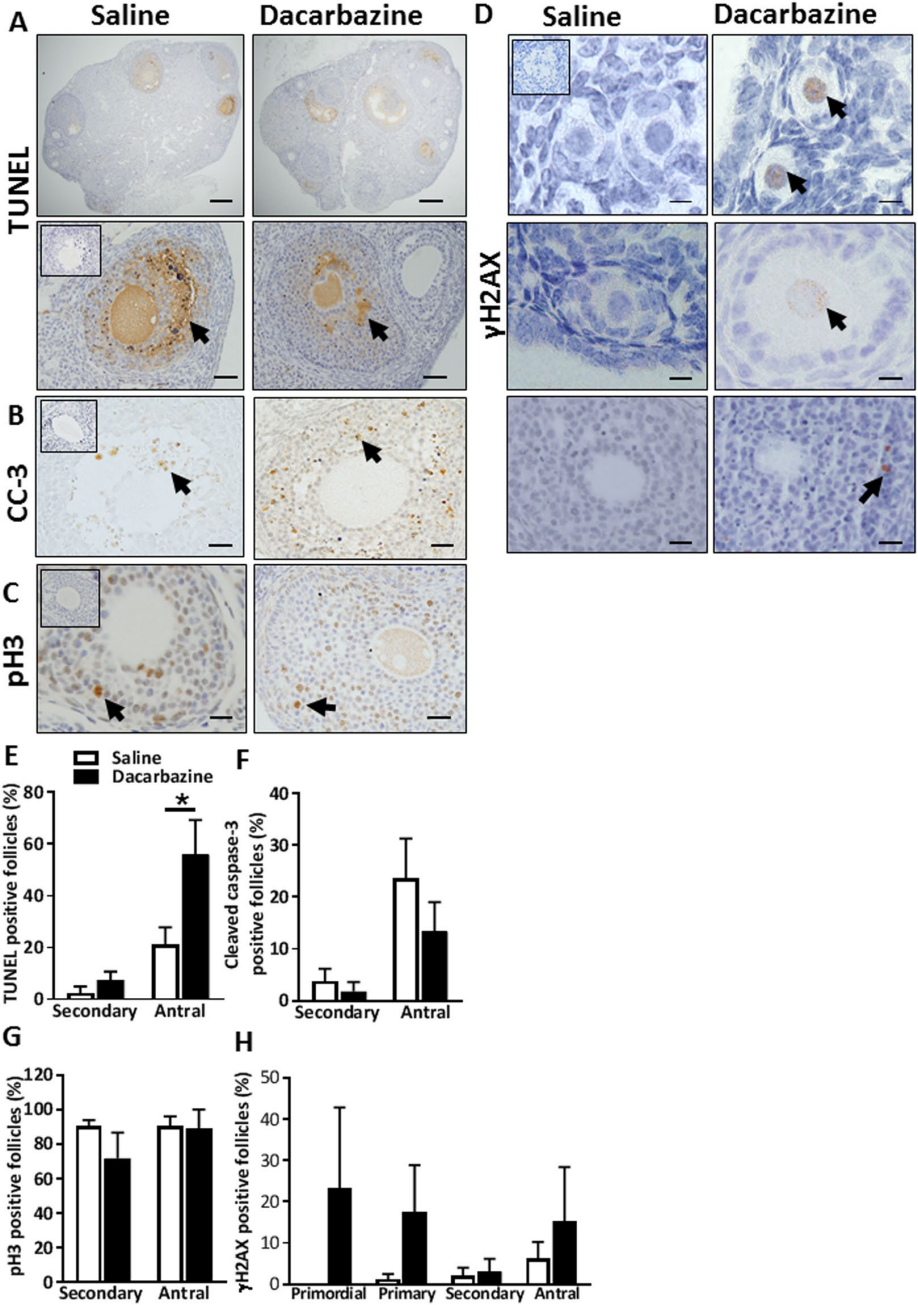
Analysis of follicle atresia, proliferation and DNA damage in 6 month old saline, or dacarbazine treated mouse ovaries at d7 + 12 h. Representative images of whole ovarian sections (upper panels) and positive follicles (brown staining, arrows, lower panels) stained for (**A**) TUNEL. Scale bars = 200 μm (upper panels) and 50 μm (lower panels). (**B**) Representative images of cleaved caspase-3 (CC-3) (Scale bars = 50 μm), (**C**) phospho-histone H3 (p H3) (Scale bars = 50 μm), and (**D**) γH2AX immunostained ovarian sections. Scale bars = 20 μm (upper panels) and 50μm (middle and lower panels). Insets are negative controls. Quantification of the proportion (%) of positive stained follicles for (**E**) TUNEL, (**F**) cleaved caspase-3, (**G**) phospho-histone H3 and (**H**) γH2AX. Data are mean ± SEM; t-test; *p < 0.05, n = 5/group.

Follicular atresia was monitored by morphological classification as above and also confirmed by TUNEL staining (Figs 7A and 8A) and cleaved caspase-3 immunostaining at the d7 + 12 h time point (Figs 7B and 8B). TUNEL and cleaved caspase-3 staining were consistently absent in primordial, transitional and primary follicles of saline and dacarbazine treated ovaries from 8 week and 6 month old mice. Staining was limited to the granulosa cells of secondary and antral follicles. There were no differences in the proportion of TUNEL-positive (Figs 7E and 8E), or cleaved caspase-3-positive secondary follicles between treatment groups at either age (Figs 7F and 8F). In line with morphological assessment of atretic antral follicles, there was a significant increase in the proportion of TUNEL-positive antral follicles in response to dacarbazine treatment versus control, in both 8 week (Fig. 7E, p < 0.05) and 6 month old mice (Fig. 8E, p < 0.05). Upon quantification of cleaved caspase-3 immunostaining, we found no difference in the percentage of positive secondary or antral follicles between treatment groups at either age (Figs 7F and 8F).

Since proliferating cells may exhibit greater sensitivity to chemotherapy, granulosa cell proliferation was monitored using pH3 staining. Positive immunostaining for pH3 was undetectable in primordial and transitional follicles and rarely present in primary follicles of all ovarian sections examined, likely due to the low mitotic index of these cells. Positive pH3 immunostaining was frequently evident in the granulosa cells of secondary and antral follicles in both dacarbazine and saline treated animals at 8 weeks (Fig. 7C) and 6 months of age (Fig. 8C). Though there were no differences in the proportions of positive follicles between treatment groups at either age (Figs 7G and 8G).

To assess whether dacarbazine induced DNA damage within follicles, γH2AX immunostaining was performed on ovarian tissue sections. Positive γH2AX localisation indicates the presence of DNA double stand breaks and the commencement of DNA damage repair. Little to no γH2AX-positive immunostaining was detected at d7 + 12 h in primordial follicle oocytes from saline control mice at 8 weeks (Figs 7D and 8H) and at 6 months of age (Figs 7D and 8H). Although there was no statistical difference, γH2AX was evident in primordial follicle oocytes from dacarbazine treated animals at both ages (Figs 7D,H and 8D,H). Similarly, there were no differences in the proportion of γH2AX-positive follicles for the other follicle classes between treatment groups, at both ages (Figs 7D,H and 8D,H).

Picrosirius red staining was performed to detect collagen deposition in the ovary. Collagen was readily detected in the mouse ovary in both 8 week and 6 month old mice (Supp. Figure 2A), although there was no treatment effect upon quantification of the staining area in either age group (Supp. Figure 2B).

## Discussion

Following a comprehensive *in vivo* animal study, we report that dacarbazine reduces the ovarian reserve of primordial follicles in mice. We also determined the comparative effects of a chemotherapeutic agent on the ovary in reproductively young, versus aged mice for the first time, highlighting that dacarbazine-mediated primordial follicle depletion is enhanced with age. Additionally, we found an immediate increase in the proportion of atretic antral follicles, and a corresponding reduction in the healthy population of antral follicles. Nevertheless, this reduction in the growing follicle population was not sufficient to physiologically disrupt normal estrous cycling, serum AMH concentrations, or ovulation, as indicated by unchanged CL numbers between treatment groups.

Our data are somewhat supported by the only previous report of the effects of dacarbazine on the ovary in mice, showing a small 16% reduction of combined primordial/primary follicle density, 14d following a single low dose, with successful pregnancy occurring in only one of four dacarbazine treated mice^15^. However, this study did not quantify absolute follicle numbers, making it impossible to interpret the ovotoxic potential of dacarbazine as a single agent. In contrast, our data have unequivocally proven that dacarbazine depletes the ovarian reserve of primordial follicles *in vivo*, in mice. This key finding directly challenges the perception that dacarbazine-containing chemotherapy protocols, such as ABVD, pose a low risk to long term female fertility^19,21^.

In the present study, the reduction in primordial follicles was accelerated in reproductively older, versus young mice (i.e. a significant reduction was observed at the 12 hour time point in older but not in younger mice until the 14 day time point). Although it should be noted that 6 month old mice are not considered to be reproductively aged, we still found that this age-mediated depletion not only occurred earlier than was detected in young mice, but we also observed a greater proportion of follicle loss in older versus young mice. To our knowledge, this is the very first comparative report of the effects of chemotherapy on the ovary between reproductively young and older mice. Given that ovarian aging has many similar characteristics in humans and mice^10^, with the exception of menopause, this finding has important clinical implications for women of advanced reproductive age receiving dacarbazine treatment, that still wish to preserve fertility. The age-related differences we observed may be attributed to a smaller primordial follicle pool in the ovary with advanced age. Alternatively, recent evidence suggests that the expression of key DNA repair factors, declines in oocytes with age in both humans and mice^31^. This may therefore impair the ability of older oocytes to repair DNA damage sustained in response to chemotherapy, although this remains to be investigated.

Menstrual cycling and AMH serum concentrations are both used clinically as indicators of ovarian damage in response to chemotherapy (reviewed^5^). Unfortunately though, there are no methods available to directly measure changes in the ovarian reserve of primordial follicles, which is the key determining factor for female fertile lifespan. Our data prove that dacarbazine depletes the ovarian reserve *in vivo*. Conversely, we found no immediate impacts on estrous cycling, serum AMH, or total number of CL, indicating normal ovulation, although this was not directly assessed. Although not significant, dacarbazine treatment led to a trend in reduced number of CL per ovary in 6 month old mice, suggesting that increased sample sizes may have been a limitation of this study for some parameters examined. Regardless, these finding suggest that in a clinical setting, it is possible that chemotherapy-induced depletion of the ovarian reserve may go undetected, until premature ovarian insufficiency and early menopause ensue. Importantly, our data contradict current clinical advice to women receiving dacarbazine treatment as part of the combined ABVD regimen, to disregard fertility preservation strategies^21^.

We sought to understand the molecular changes underlying dacarbazine-mediated follicle loss in ovarian tissues using immunohistochemical and histological analyses. We analysed tissues collected at the d7 + 12 h time point to evaluate potential immediate effects of dacarbazine on follicle atresia, proliferation and DNA damage. Despite our results showing a reduction in primordial follicles after dacarbazine exposure, TUNEL and cleaved caspase-3 analysis did not detect evidence of increased apoptosis within primordial follicles at d7 + 12 h. This finding is not unique, with previous studies highlighting the inability of current markers to detect apoptotic primordial follicles, even during periods of dramatic follicle loss^27,28,32^. This affirms the importance of accurate quantification of absolute primordial follicle numbers. Furthermore, loss of primordial follicles in the absence of increased transitional or primary follicles, indicate that the reduction in ovarian reserve is likely mediated by direct primordial follicle damage and death, rather than transition into the growing follicle population. In contrast to our findings in primordial follicles, and in line with morphological quantification, TUNEL staining faithfully detected a significant increase in the proportion of atretic antral follicles in dacarbazine treated ovaries, versus controls. Overall, however, even though dacarbazine-mediated depletion of healthy antral follicles 14d following final treatment, unchanged CL number and estrous cyclicity suggests that sufficient antral follicles remained to support ovulation.

DNA double strand breaks are commonly induced by chemotherapeutic agents, resulting in activation of the DNA damage response and phosphorylation of histone H2AX^33^. At 12 h, we observed γH2AX immunostaining in primordial follicles from dacarbazine treated mice, but not in controls, although, due to high variability, this was not statistically significant. Importantly though, an earlier study found induction of chromosomal aberrations by dacarbazine in both somatic and germ cells of male mice, which ultimately resulted in genetic mutations in offspring^23^. However, this remains to be thoroughly investigated in females.

Dacarbazine is associated with lung fibrosis in human patients^34^, therefore, we examined ovarian tissue fibrosis in dacarbazine treated animals using Picrosirius red staining to detect collagen deposition^30^. Collagen was readily detected in the mouse ovary in both 8 week and 6 month old mice, however, there was no treatment effect upon quantification of the staining area in either age group at the d7 + 12 h time point. It is possible that a dacarbazine-mediated increase in collagen accumulation may occur beyond this time point, although this was not examined.

Our findings overall highlight the importance of rigorous preclinical research using animal models to determine the potential for different chemotherapeutics to affect the ovary in humans. The mouse is a physiologically relevant model for studying the impacts of chemotherapeutic agents on ovarian follicle populations. In particular, with regard to dacarbazine, similarities in cytochrome P450 activity between humans and mice^35^, suggest that the metabolism of dacarbazine is likely comparable between the two species. Importantly, the dacarbazine treatment regimen used in this study is representative of a single chemotherapy cycle. Alarmingly, the ovotoxic effects of dacarbazine were observed as soon as only one week following treatment. Increases in total cumulative dose are known to increase the severity of chemotherapy-induced ovarian damage^36^, an important factor to consider, given that in a clinical setting, cancer patients receive multiple doses/cycles of chemotherapy (e.g. between 2 and 8 cycles of ABVD are often given) and therefore the impact of dacarbazine on the ovarian reserve of primordial follicles may be further amplified in women, than reported here.

Dacarbazine is one of four components of ABVD chemotherapy regimen, which is commonly administered to young cancer patients. Using human ovarian biopsy samples, one recent study found an increase in primordial follicle density following ABVD chemotherapy, compared to an untreated, or OEPA-COPDAC treated group of women^19^. Given the limitations of obtaining primary human tissue, follicle density is often reported as a surrogate measure for absolute follicle number in the ovary. However, this quantification method does not account for the unequal distribution of follicles throughout the ovary, or changes in ovarian volume, and often only samples a small biopsy or fraction of the ovary, and thus lacks the accuracy achievable with stereological techniques employed in the present study. Indeed, we found that measures of absolute follicle quantification and follicle density, did not correspond in mouse ovaries. We found no differences in follicle density between treatment groups, across all follicle classes and at both time points and ages that were examined. Although our study was limited to the administration of a single chemotherapeutic agent, our data indicate a gonadotoxic effect of dacarbazine, which is unlikely to lead to an increase in the primordial follicle population in the human ovary as recently reported^19^, though does not preclude that possibility that the ovary may respond differently when exposed to multiple drugs simultaneously. Notably, in order to definitively confirm whether dacarbazine impairs female fertility, a thorough breeding study is required to determine the impact on fertile lifespan, oocyte quality and offspring health.

In summary, dacarbazine administration reduced the ovarian reserve of primordial follicles in mice and depletion was enhanced with age. We observed an acute reduction in the antral follicle population in response to dacarbazine, although AMH serum levels and estrous cycling were unaltered. These findings have important clinical implications, since female cancer patients may present with normal menstrual cycles and AMH levels following chemotherapy treatment, despite having a diminished ovarian reserve. Since reduced ovarian reserve can ultimately result in early loss of fertility and premature menopause, counselling and fertility preservation should be considered for young female patients prior to dacarbazine treatment.

## Electronic supplementary material

Supplementary Data
