# Supplementary material for: Dacarbazine depletes the ovarian reserve in mice and depletion is enhanced with age
Source: Sci Rep. 2018 Apr 25;8:6516. doi: 10.1038/s41598-018-24960-5 (PMC5917018; doi:10.1038/s41598-018-24960-5)

**Supplementary Data**

**Dacarbazine depletes the ovarian reserve in mice and depletion is enhanced with age.**

**Amy L. Winship, Monika Bakai, Urooza Sarma, Seng H. Liew and Karla J. Hutt.**


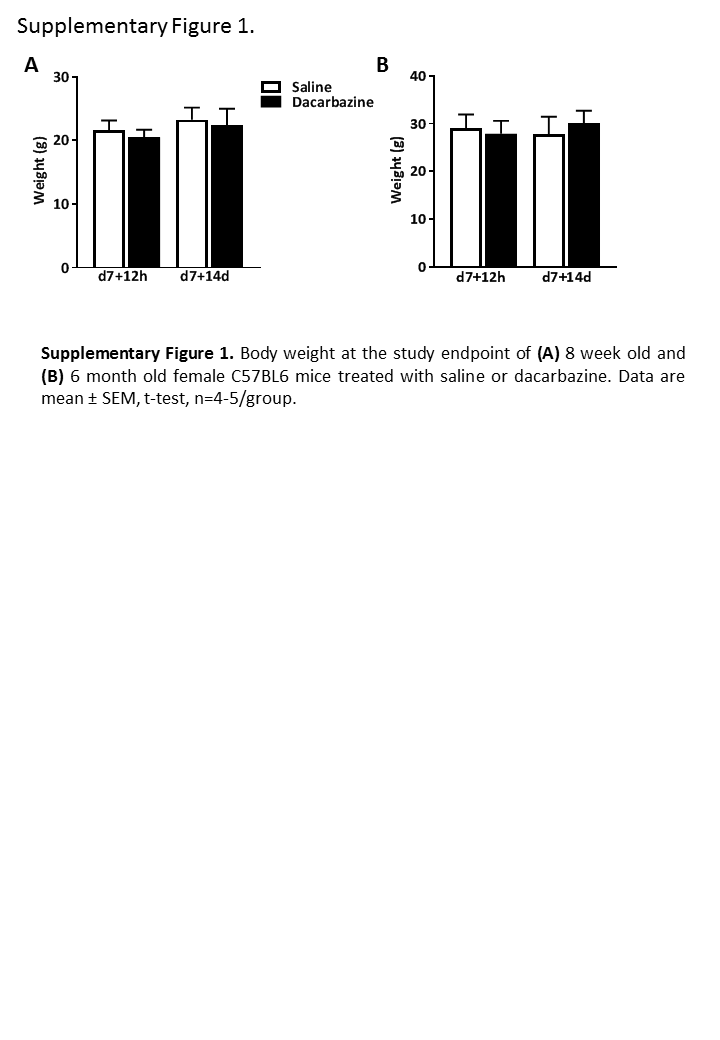


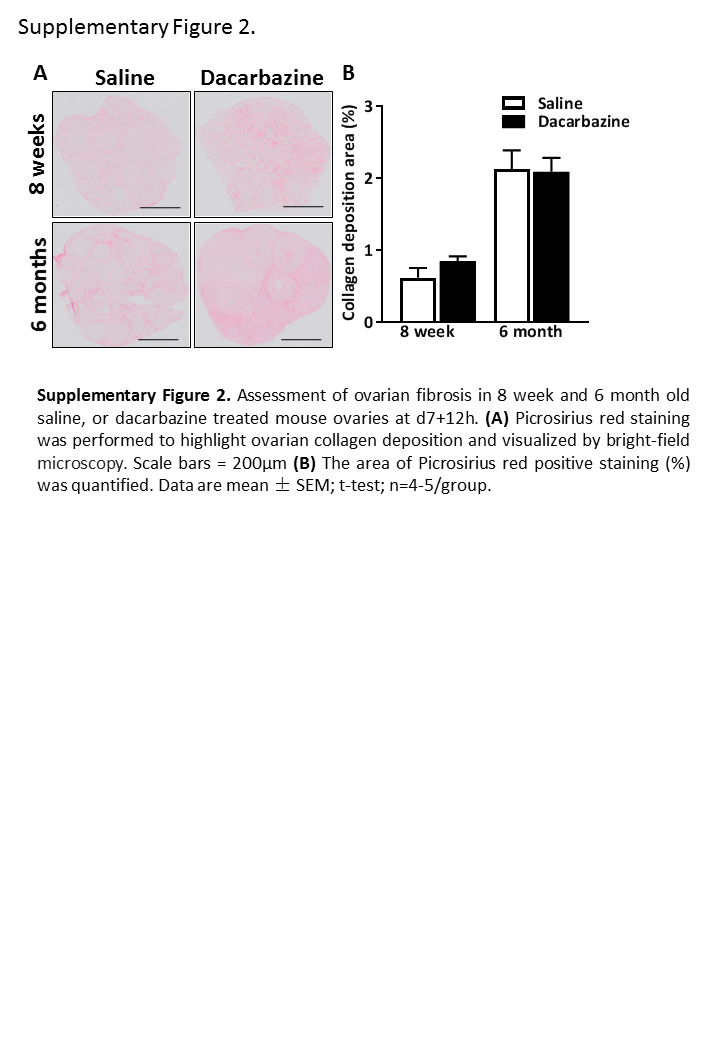

Supplement: Supplementary file 1 — Supplementary Data [file 41598_2018_24960_MOESM1_ESM.docx]
